# Sepsis and disseminated intravascular coagulation

**DOI:** 10.1186/s40560-016-0149-0

**Published:** 2016-03-23

**Authors:** Kohji Okamoto, Toshihisa Tamura, Yusuke Sawatsubashi

**Affiliations:** Department of Surgery, Center for Gastroenterology and Liver Disease, Kitakyushu City Yahata Hospital, 4-18-1 Nishihon-machi, Yahatahigashi-ku, Kitakyushu 805-8534 Japan; Department of Surgery 1, School of Medicine, University of Occupational & Environmental Health, 1-1 Iseiogaka, Yahatanishi-ku, Kitakyushu 807-8555 Japan

**Keywords:** Sepsis, Disseminated intravascular coagulation (DIC), HMGB1, Antithrombin, Thrombomodulin

## Abstract

Sepsis is frequently complicated by coagulopathy and, in about 35 % of severe cases, by disseminated intravascular coagulation (DIC). In Japan, aggressive treatment of septic DIC is encouraged using antithrombin and recombinant thrombomodulin. The macrophages, monocytes, and neutrophils are a source of TF and participate in the direct activation of the coagulation cascade in the early phases of sepsis. And activated factor X (FXa), which is involved in hemostasis, thrombogenesis, inflammation, and cellular immune responses, induces TF expression in human peripheral monocytes and, conversely, that inhibition of FXa activity reduces TF expression. Both inflammation and coagulation play an important role in DIC due to sepsis. In addition to inflammatory cytokines (TNF-α, IL-1 and so on), HMGB1 has recently been shown to mediate the lethal late phase of sepsis and caused coagulopathy. TM not only binds HMGB1 but also aids the proteolytic cleavage of HMGB1 by thrombin. There have been many reports of the efficacy of recombinant TM and antithrombin for treatment of septic DIC from Japan. Further investigation of the efficacy of recombinant TM and AT in countries other than Japan, as well as the monitoring of medical costs incurred during hospitalization, will help validate the use of TM and AT for treatment of septic DIC.

## Introduction

Sepsis is a clinical syndrome defined as a systemic response to infection. It is frequently complicated by coagulopathy [1] and, in about 35 % of severe cases, by disseminated intravascular coagulation (DIC) [2–4]. In the European Union and the USA, the 2012 guidelines of the Surviving Sepsis Campaign do not recommend treatment for septic DIC [5, 6]. In contrast, in Japan, aggressive treatment of septic DIC is encouraged [7–9]. It is not an exaggeration to state that Japan is one of the countries that most effectively treats patients with septic DIC. In this article, we review the mechanisms that underlie the interaction between sepsis and DIC and, by highlighting our findings, the effects of sepsis on the coagulation system.

## Review

### Sepsis-induced DIC

During sepsis, inflammation diffusely activates the coagulation system, consuming multiple clotting factors and resulting in DIC [10, 11]. In systemic inflammatory response syndromes caused by infection, both perturbed endothelial cells and activated mononuclear cells produce proinflammatory cytokines that promote coagulation [12, 13]. Proteins expressed on these cells initiate coagulation. Thrombin elicits the production of monocyte chemoattractant protein 1 and interleukin (IL)-6 in monocytes, fibroblasts, and mesothelial cells, and the production of IL-6 and IL-8 in vascular endothelial cells by interacting with protease-activated receptors (PARs) 1, 3, and 4. Via PAR 2, factor Xa, and the tissue factor-VIIa complex also upregulate IL-6 and IL-8 in vascular endothelial cells [14–16]. In addition, the inhibition of physiologic anticoagulant mechanisms and fibrinolysis by endothelial cells causes intravascular fibrin deposition.

Initiation of the extrinsic coagulation protease cascade requires tissue factor (TF), a 47-KDa transmembrane glycoprotein [17]. We reported that macrophages, monocytes, and neutrophils are a source of TF in sepsis animal models and participate in the direct activation of the coagulation cascade in the early phases of sepsis [18–20]. We also showed that activated factor X (FXa), which is involved in hemostasis, thrombogenesis, inflammation, and cellular immune responses, induces TF expression in human peripheral monocytes and, conversely, that inhibition of FXa activity reduces TF expression in an experimental model of rat endotoxemia [21]. Our results indicate that FXa directly modulates TF expression and that both inflammation and coagulation play an important role in DIC due to sepsis. Development of a procoagulant state in sepsis, due to aberrant expression of tissue factor (TF) and sharp decrease of its major inhibitor tissue factor pathway inhibitor (TFPI), could lead to microthrombotic organ failure [22]. TFPI is a major inhibitor of the TF-FVIIa-initiated coagulation in vivo. Tang et al. [22] and Gando S et al. [23] suggested that during early sepsis, the available TFPI might not adequately balance the increased TF-dependent coagulation activation. Moreover Tang et al. suggested that plasmin might be partly responsible for proteolytic degradation of TFPI in the early stages of sepsis.

**Fig. 1:**
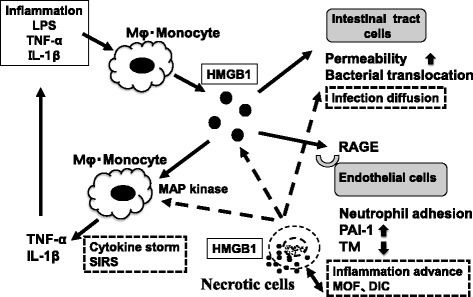
The various functions of HMGB1 in sepsis. HMGB1 is actively secreted from macrophages and monocytes, which are activated by inflammatory cytokines, and it is also passively released from necrotic cells. HMGB1 may then cause activation of phagocytic cells, resulting in production of pro-inflammatory mediators and chemokines. HMGB1 binds to RAGE on endothelial cells. And endothelial cells express RAGE, adhesion molecules, TNF-α, chemokines, PAI-1, and promote down regulation of TM. *RAGE* receptor for advanced glycation end-products, *IL* interleukin, *TNF* tumor necrosis factor, *PAI-1* plasminogen activator inhibitor-1, *DIC* disseminated intravascular. Coagulation, *SIRS* systemic inflammatory response syndrome, *MAP* mitogen-activated protein

In addition to inflammatory cytokines, other factors have recently been shown to mediate the lethal late phase of sepsis; these factors include tumor necrosis factor (TNF)-α, IL-1, high-mobility group box-1 (HMGB1) protein, and nuclear architectural chromatin-binding protein [24]. HMGB1 is secreted by activated monocytes and macrophages [25] and released from necrotic or damaged cells [26]. Extracellular HMGB1 mediates cell-to-cell signaling and activates proinflammatory pathways [27]. When released into the extracellular space, it elicits the production of inflammatory cytokines [25], which further augment the release of HMGB1 into the extracellular space [28]. The recent published findings by Lu et al. [29] demonstrate that hyperacetylated HMGB1 is a novel biomarker for pyroptosis, though necrosis-induced HMGB1 release is not acetylated. Moreover, tissue damage induces the release of HMGB1 with all-cysteines reduced, whereas this form of HMGB1 does not stimulate cytokine release; it recruits leukocytes to the site of injury. And during infection or later stage of injury, HMGB1 released is acetylated or disulfide-bonded, and it stimulates cytokine release [30]. The various functions of HMGB1 are shown in Fig. 1.

**Fig. 2:**
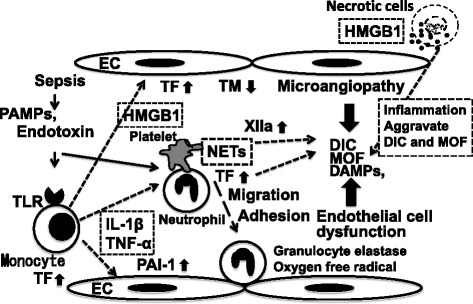
A mechanism of DIC and MOF due to sepsis. When the pathogen-associated molecular patterns (PAMPs) (for example, endotoxin) and damage-associated molecular patterns (DAMPs) act on monocytes via TLR and on  neutrophils, a reactivated monocyte produce TF, various inflammatory cytokines, and HMGB1, and moreover, detection of PAMPs and DAMPs trigger neutrophil extracellular traps (NETs) release by neutrophils, promoting immunothrombosis. The uncontrolled immunothrombosis may lead to disseminated intravascular coagulation. And HMGB1 acts on EC and promotes upregulation of TF and downregulation of TM from EC, resulting endothelial cell injury, and microcirculation disorder develops DIC and MOF. *TF* tissue factor, *TM* thrombomodulin, *TLR* Toll-like receptor, *IL-1β* interleukin-1β, *TNF-α* tumor necrosis factor-α, *EC* endothelial cell, *HMGB1* high-mobility group box protein 1, *PAI* plasminogen activator inhibitor, *MOF* multiple organ failure, *NETs* neutrophil extracellular traps

Recently, PAMPs and DAMPs in early phase of sepsis trigger tissue factor expression on monocytes and neutrophil extracellular trap (NET) release by neutrophils, promoting immunothrombosis. Although immunothrombosis plays a role in early host defense against bacterial dissemination, uncontrolled immunothrombosis may also lead to DIC [31]. Besides, recent studies have identified histones, the most abundant proteins in the nucleus, as a new class of DAMPs [32–35]. Extracellular histones promote neutrophil migration, platelet aggregation, and endothelial cell death [32, 36, 37]. Histones have been detected in the plasma of mice, baboons, and human patients with sepsis and trauma, and the total concentration of histones can reach 70, with that of histone H3 reaching 15 μg/ml [32, 38]. Nakahara et al. suggested that extracellular histones cause massive thromboembolism associated with consumptive coagulopathy, which is diagnostically indistinguishable from DIC and that rTM binds to histones and neutralizes the prothrombotic action of histones [39]. A mechanism of DIC and MOF due to sepsis are shown in Fig. 2.

**Fig. 3:**
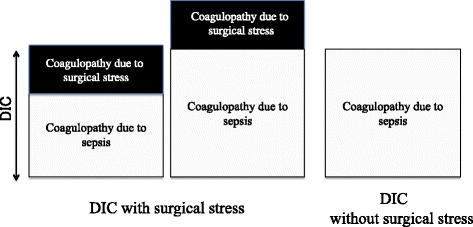
Effect of surgical stress for coagulopathy (DIC) due to infection. If the severity of the infectious disease is the same, coagulopathy of infectious disease in surgically patients is increased by addition of the coagulation disorder due to surgical stress. In the treatment of infection control, the surgeons and intensivists must take that coagulopathy of the surgical stress deteriorates DIC temporarily into consideration

Moreover, if the severity of the infectious disease is the same, coagulopathy of infectious disease in surgically patients is increased by addition of the coagulation disorder due to surgical stress (Fig.3). In treatment of basic disease, the surgeons and intensivists must take that coagulopathy of the surgical stress deteriorates DIC temporarily into consideration.

### Diagnostic criteria of septic DIC

Different diagnostic criteria of septic DIC have been established by the International Society on Thrombosis and Haemostasis [40], the Japanese Ministry of Health, Labor and Welfare (JMHLW) [41], and the Japanese Association of Acute Medicine (JAAM) [42].

Although the criteria of the JAAM are the most specific for septic DIC [42, 43], a prospective study in Japan found no significant differences in the odds ratios for prediction of DIC outcomes calculated on the basis of these three diagnostic criteria [44]. As the mortality rate of DIC is still high, early diagnosis and treatment are required.

### Laboratory tests

Screening assays (global coagulation tests) using scoring parameters, such as prothrombin time, fibrinogen level, platelet count, and levels of fibrin-related markers, provide important information about the degree of coagulation factor activation and consumption.

Examination of DIC scores (based on the JMHLW criteria) at the beginning of DIC treatment showed that greater treatment efficacy was achieved in pre-DIC than in DIC patients [45]. Outcome worsened as the DIC score increased, thus suggesting that both early diagnosis and early treatment of DIC are important. To define the pre-DIC state, we prospectively evaluated global coagulation tests, hemostatic molecular markers, and the onset of DIC within a week after registration [46]. The levels of D-dimer and FMC were significantly lower in patients with pre-DIC than in those without DIC, whereas there were no significant differences in the levels of thrombin-antithrombin complex (TAT), plasmin-α2plasmin inhibitor complex (PIC), antithrombin (AT), and thrombomodulin (TM). However, no markers that provided an appropriate cutoff value for differentiating between “pre-DIC” and “without DIC” (as do DIC scores) were identified.

### Treatment of septic DIC

Common sense dictates that administration of an antibiotic that specifically targets the infection is the most important therapy in septic DIC. After administering antibiotics, surgical drainage at the infection site should be performed as soon as possible. Physicians should first administer treatment for the underlying disease when sepsis is diagnosed [4, 8].

### Antithrombin

AT is a single-stranded glycoprotein with a molecular weight of ca. 59,000. It is synthesized in the liver and inhibits the activity of thrombin and activated factors X, IX, VII, XI, and XII [47]. Extensive clinical studies have been performed in patients with severe sepsis [48–53] to determine the appropriate dose of AT. Twenty-eight days of AT treatment did not improve the survival rate in the KyberSept trial [48], which was a multicenter, double-blind phase III study that included 2314 patients with severe sepsis (a total of 30,000 IU of AT was administered over 4 days). However, in a subgroup analysis, an improvement in the survival rate on day 90 was observed in patients not receiving concomitant heparin treatment; this finding agrees with the results of previous phase II studies supporting the efficacy of AT [54–58]. A recent Japanese study by Iba et al. [59] used a nonrandomized, multi-institutional, post-marketing survey to determine the optimal AT dose for treating septic DIC. They reported survival rates of 65.2 % in patients receiving 1500 IU/day and 74.7 % in patients receiving 3000 IU/day. A logistic regression analysis showed that the higher dose (3000 IU/day) was associated with a better survival outcome [59]. A second survey, in which the baseline AT levels in patients with septic DIC were less than 40 %, showed a significantly higher rate of DIC resolution and a better survival outcome in patients receiving 3000 IU/day compared with those receiving 1500 IU/day [60]. The ratio of bleeding events in the two groups was not significantly different.

We conducted a prospective, randomized, controlled multicenter trial for DIC patients with sepsis and AT levels of 50 to 80 % to test the hypothesis that concentrated administration of AT improves DIC, resulting in faster recoveries and better outcomes [61]. Patients receiving AT for 3 days had significantly lower DIC scores and higher recovery rates than did those who did not receive AT. This finding suggests that moderate doses of AT (30 IU/kg per day) improve DIC scores, thereby increasing the recovery rate without any risk of bleeding in patients with septic DIC.

Tagami et al. [62] performed an analysis using information collected from a nationwide administrative database in Japan. Patients with severe pneumonia and DIC (n=9075) were divided into an AT group (n=2663) and a control (no AT) group (n=6412). Propensity score matching created a matched cohort of 2194 paired patients who did or not receive AT treatment. The 28-day mortality rate was 9.9 % lower in the AT group than in the control group. Multiple logistic regression analyses showed an association between AT use and the 28-day mortality rate (adjusted odds ratio, 0.85).

### Heparin

The British guidelines recommend the use of unfractionated heparin (UFH) because of its short half-life and availability of antagonists, especially in patients at a high risk of bleeding. Japanese guidelines indicate a preference for low molecular weight heparin because it proved superior in improving coagulation abnormalities and caused fewer hemorrhagic adverse events in a randomized controlled trial (RCT) conducted in DIC [63]. In the HETRASE (A Randomized Clinical Trial of Unfractioned Heparin for Treatment of Sepsis) study [64], the results of which were reported after publication of the guidelines, and the efficacy of UFH for sepsis was denied. Zarychanski R et al. [65] reported that the risk hazard ratio for death associated with the use of heparin in septic patients was 0.88 (95 % confidence interval (CI), 0.77**–**1.00; *I*^2^ = 0 %). In addition, Wang et al. [66] also reported a decreased mortality associated with heparin use (odds ratio = 0.656, 95 % CI = 0.562**–**0.765, *P* < 0.0001). Moreover, Iba et al. [67] reported that both UFH and LMWH attenuated the toxicity of histone H3, in vivo as well as in vitro, and that the effects of heparins shown in ex vivo study were independent of their anticoagulant effect. They suggested that the administration of heparin could become a treatment of choice for patients suffering from severe sepsis.

### Thrombomodulin

TM is an endothelial anticoagulant cofactor that plays an important role in the regulation of intravascular coagulation [68]. It accelerates the thrombin-catalyzed conversion of protein C to activated protein C, which inhibits monocyte and macrophage activation [69, 70] and consequently suppresses the production of inflammatory cytokines such as TNF-α and IL-1β [70]. In addition, recent studies have shown that TM binds to HMGB1 to prevent its interaction with the receptors for advanced glycation end-products [71]. We reported that TM not only binds HMGB1 but also aids the proteolytic cleavage of HMGB1 by thrombin [72]. These findings highlight the novel anti-inflammatory actions of TM.

**Fig. 4:**
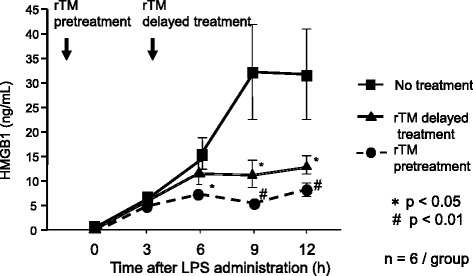
Effect of rTM on the plasma levels of HMGB1. Temporal changes in plasma HMGB1 concentrations after injection of lipopolysaccharide (LPS). Rats were given saline plus LPS (*closed squares*); pretreatment of recombinant human soluble thrombomodulin (*rTM*), LPS plus saline (*closed circles*); or saline, LPS plus delayed treatment of rTM (*closed triangles*). All data represent the mean and SEM (*n* = 6 per group). [73] *P < 0.05 (vs. the LPS group). ^#^ *P* < 0.01 (vs. the LPS group). *rTM* recombinant thrombomodulin

We investigated the effects of soluble recombinant human TM on the production of inflammatory cytokines and the plasma level of HMGB1 in an experimental endotoxemia model [73]. Endotoxemia was induced in rats via a bolus intravenous injection of 4 mg/kg lipopolysaccharide (LPS). Recombinant TM (1 mg/kg) was administered as a bolus injection 30 min before or 4 h after LPS. LPS increased the plasma levels of TNF-α and IL-1β, which peaked at 1 and 3 h, respectively, and over time, the plasma levels of HMGB1. Even when its administration was delayed, recombinant TM markedly inhibited the LPS-induced increase in plasma levels of HMGB1 (Fig. 4) and the thrombin-AT complex, as well as the increase in liver dysfunction and mortality. The use of recombinant TM may therefore be beneficial for treatment of septic patients.

In a Japanese phase III randomized control trial (RCT) in which 227 DIC patients with 125 hematological malignancies and 102 infections (sepsis) received recombinant TM or unfractionated heparin (UFH), the rate of resolution of DIC was 66.1 and 49.9 %, respectively [74]. The rate of disappearance of bleeding was 35.2 % in the recombinant TM group and 20.9 % in the UFH group, and the 28-day mortality rate was 28.0 and 34.6 %, respectively. In an analysis of 80 patients with infectious DIC, the rate of resolution of DIC was 63.2 % in the UFH group and 73.2 % in the recombinant TM group [75]. In an international phase II RCT of 750 septic patients with suspected DIC, the 28-day mortality rate was 17.8 % in the recombinant TM group and 21.6 % in the placebo group [76]; there was a tendency toward a low rate in the TM group, although the difference was not significant (*P* = 0.273). An international phase III clinical trial evaluating the efficacy of TM in patients with severe sepsis and coagulopathy is ongoing in the USA, South America, Asia, Australia, the European Union, and other countries (https://clinicaltrials.gov/ct2/show/NCT01598831?term=ART-123&rank=2).

On the other hand, Tagami et al. [77] found that recombinant TM was not an effective treatment for sepsis-associated DIC following severe pneumonia. This conclusion was based on propensity scores and an instrumental variable analysis of information obtained from the Japanese Diagnosis Procedure Combination (JDPC) inpatient database, a nationwide administrative database. No significant difference in the 28-day mortality rate was documented between the two groups in a propensity-matched analysis.

We also evaluated the efficacy of recombinant TM for DIC using the JDPC database [78–80]. We found that the frequency of use of AT, heparin, and protease inhibitors decreased from 2010 to 2012 in Japan, while that of recombinant TM significantly increased (25.1, 43.1, and 56.8 % in 2010, 2011, and 2012, respectively; *P* < 0.001). Logistic regression analysis showed that the study period was associated with the use of recombinant TM in patients with DIC. The odds ratio (OR) was 2.34 (95 % confidence interval [CI], 2.12–2 to 58; *P* < 0.001) in 2011 compared with 4.34 (95 % CI, 3.94–4.79; *P* < 0.001) in 2012. Large hospital size was the most significant factor associated with the use of recombinant TM in patients with DIC (OR, 3.14; 95 % CI, 2.68–3.66; *P* < 0.001). The use of recombinant TM has dramatically increased, and a large hospital size was significantly associated with increased use from 2010 to 2012 in Japan. We found no significant difference in the in-hospital mortality rate between patients receiving AT and recombinant TM. However, the administration of recombinant TM was significantly associated with lower hospitalization times and medical costs during hospitalization.

## Conclusions

This review discussed the mechanisms that underlie the interaction between sepsis and DIC and the effects of sepsis on the coagulation system, as highlighted by our data. Further investigation of the efficacy of recombinant TM and AT in countries other than Japan, as well as the monitoring of medical costs incurred during hospitalization, will help validate the use of TM and AT for treatment of septic DIC.

## Abbreviations

AT: antithrombin
CI: confidence interval
DAMPs: damage-associated molecular patterns
DIC: disseminated intravascular coagulation
FXa: activated factor X
HMGB1: high-mobility group box-1
IL: interleukin
JAAM: Japanese Association of Acute Medicine
JDPC: Japanese Diagnosis Procedure Combination
JMHLW: Japanese Ministry of Health, Labor and Welfare
LPS: lipopolysaccharide
OR: odds ratio
PAMPs: pathogen-associated molecular patterns
PAR: protease-activated receptor
PIC: plasmin-α2plasmin inhibitor complex
RCT: randomized control trial
TAT: thrombin-antithrombin complex
TF: tissue factor
TM: thrombomodulin
TNF-α: tumor necrosis factor
UHF: unfractionated heparin

## References

[1] Kinasewitz GT, Yan SB, Basson B, Comp P, Russell JA, Cariou A, Um SL, Utterback B, Laterre PF, Dhainaut JF, PROWESS Sepsis Study Group (2004). Universal changes in biomarkers of coagulation and inflammation occur in patients with severe sepsis, regardless of causative micro-organism [ISRCTN74215569]. Crit Care.

[2] Levi M, ten Cate H (1999). Disseminated intravascular coagulation. N Engl J Med.

[3] Bakhtiari K, Meijers JC, de Jonge E, Levi M (2004). Prospective validation of the International Society of Thrombosis and Haemostasis scoring system for disseminated intravascular coagulation. Crit Care Med.

[4] Dhainaut JF, Yan SB, Joyce DE, Pettilä V, Basson B, Brandt JT, Sundin DP, Levi M (2004). Treatment effects of drotrecogin alfa (activated) in patients with severe sepsis with or without overt disseminated intravascular coagulation. J Thromb Haemost.

[5] Dellinger RP, Levy MM, Rhodes A, Annane D, Gerlach H, Opal SM, Sevransky JE, Sprung CL, Douglas IS, Jaeschke R, Osborn TM, Nunnally ME, Townsend SR, Reinhart K, Kleinpell RM, Angus DC, Deutschman CS, Machado FR, Rubenfeld GD, Webb S, Beale RJ, Vincent JL, Moreno R (2013). Surviving Sepsis Campaign Guidelines Committee including The Pediatric Subgroup. Surviving Sepsis Campaign: international guidelines for management of severe sepsis and septic shock, 2012. Intensive Care Med.

[6] Dellinger RP, Levy MM, Rhodes A, Annane D, Gerlach H, Opal SM, Sevransky JE, Sprung CL, Douglas IS, Jaeschke R, Osborn TM, Nunnally ME, Townsend SR, Reinhart K, Kleinpell RM, Angus DC, Deutschman CS, Machado FR, Rubenfeld GD, Webb SA, Beale RJ, Vincent JL, Moreno R, Surviving Sepsis Campaign Guidelines Committee including the Pediatric Subgroup (2013). Surviving sepsis campaign: international guidelines for management of severe sepsis and septic shock: 2012. Crit Care Med.

[7] Oda S, Aibiki M, Ikeda T, Imaizumi H, Endo S, Ochiai R, Kotani J, Shime N, Nishida O, Noguchi T, Matsuda N, Hirasawa H (2014). Sepsis Registry Committee of The Japanese Society of Intensive Care Medicine. The Japanese guidelines for the management of sepsis. J Intensive Care.

[8] Wada H, Asakura H, Okamoto K, Iba T, Uchiyama T, Kawasugi K, Koga S, Mayumi T, Koike K, Gando S, Kushimoto S, Seki Y, Madoiwa S, Maruyama I, Yoshioka A (2010). Japanese Society of Thrombosis Hemostasis/DIC subcommittee: expert consensus for the treatment of disseminated intravascular coagulation in Japan. Thromb Res.

[9] Wada H, Okamoto K, Iba T, Kushimoto S, Kawasugi K, Gando S, Madoiwa S, Uchiyama T, Mayumi T, Seki Y, Japanese Society of Thrombosis Hemostasis/DIC subcommittee (2014). Addition of recommendations for the use of recombinant human thrombomodulin to the “Expert consensus for the treatment of disseminated intravascular coagulation in Japan”. Thromb Res.

[10] Levi M, van der Poll T (2010). Inflammation and coagulation. Crit Care Med.

[11] O’Brien M (2012). The reciprocal relationship between inflammation and coagulation. Top Companion Anim Med.

[12] Levi M, van der Poll T, ten Cate H, van Deventer SJ (1997). The cytokine-mediated imbalance between coagulant and anticoagulant mechanisms in sepsis and endotoxemia. Eur J Clin Invest.

[13] Itoh H, Okamoto K (2002). The mechanism behind coagulation disorders and organ dysfunction due to abdominal sepsis. J Abdomin Emerg Med.

[14] Johnson K, Choi Y, DeGroot E, Samuels I, Creasey A, Aardwn I (1998). Potential mechanism for a proinflammatory vascular cytokine response to coagulation activation. J Immunol.

[15] Johnson K, Aarden L, Choi Y, De Groot E, Creasey A (1996). The proinflammatory cytokine response to coagulation and endotoxin in whole blood. Blood.

[16] Esmon CT (2000). Does inflammation contribute to thrombotic events?. Haemostasis.

[17] Camerer E, Kolsto AB, Prydz H (1996). Cell biology of tissue factor, the principal initiator of blood coagulation. Thromb Res.

[18] Higure A, Okamoto K, Hirata K, Todoroki H, Nagafuchi Y, Takeda S, Katoh H, Itoh H, Ohsato K, Nakamura S (1996). Macrophages and neutrophils infiltrating into the liver are responsible for tissue factor expression in a rabbit model of acute obstructive cholangitis. Thromb Haemost.

[19] Todoroki H, Nakamura S, Higure A, Okamoto K, Takeda S, Nagata N, Itoh H, Ohsato K (2000). Neutrophils express tissue factor in a monkey model of sepsis. Surgery.

[20] Nakamura S, Imamura T, Okamoto K (2004). Tissue factor in neutrophils: yes. J Thromb Haemost.

[21] Akahane K, Okamoto K, Kikuchi M, Todoroki H, Higure A, Sugiyama T, Kitahara K, Takeda S, Itoh H, Ohsato K (2001). Inhibition of factor Xa suppresses the expression of tissue factor in human monocytes and lipopolysaccharide induced endotoxemia in rats. Surgery.

[22] Tang H, Ivanciu L, Popescu N, Peer G, Hack E, Lupu C, Taylor FB, Lupu F (2007). Sepsis-induced coagulation in the baboon lung is associated with decreased tissue factor pathway inhibitor. Am J Pathol.

[23] Gando S, Kameue T, Morimoto Y, Matsuda N, Hayakawa M, Kemmotsu O (2002). Tissue factor production not balanced by tissue factor pathway inhibitor in sepsis promotes poor prognosis. Crit Care Med.

[24] Wang H, Yang H, Czura CJ, Sama AE, Tracey KJ (2001). HMGB1 as a late mediator of lethal systemic inflammation. Am J Respir Crit Care Med.

[25] Wang H, Bloom O, Zhang M, Vishnubhakat JM, Ombrellino M, Che J, Frazier A, Yang H, Ivanova S, Borovikova L, Manogue KR, Faist E, Abraham E, Andersson J, Andersson U, Molina PE, Abumrad NN, Sama A, Tracey KJ (1999). HMG-1 as a late mediator of endotoxin lethality in mice. Science.

[26] Scaffidi P, Misteli T, Bianchi ME (2002). Release of chromatin protein HMGB1 by necrotic cells triggers inflammation. Nature.

[27] Schmidt AM, Yan SD, Yan SF, Stern DM (2001). The multiligand receptor RAGE as a progression factor amplifying immune and inflammatory responses. J Clin Invest.

[28] Wang H, Vishnubhakat JM, Bloom O, Zhang M, Ombrellino M, Sama A, Tracey KJ (1999). Proinflammatory cytokines (tumor necrosis factor and interleukin 1) stimulate release of high mobility group protein-1 by pituicytes. Surgery.

[29] Lu B, Nakamura T, Inouye K, Li J, Tang Y, Lundbäck P, Valdes-Ferrer SI, Olofsson P, Kalb T, Roth J, Zou Y, Erlandsson-Harris H, Yang H, Ting JP, Wang H, Andersson U, Antoine DJ, Chavan SS, Hotamisligil GS, Tracey KJ (2012). Novel role of PKR in inflammasome activation and HMGB1 release. Nature.

[30] Yang H, Antoine DJ, Andersson U, Tracey KJ (2013). The many faces of HMGB1: molecular structure-functional activity in inflammation, apoptosis, and chemotaxis. J Leukoc Biol.

[31] Ito T (2014). PAMPs and DAMPs as triggers for DIC. J Intensive Care.

[32] Xu J, Zhang X, Pelayo R, Monestier M, Ammollo CT, Semerano F, Taylor FB, Esmon NL, Lupu F, Esmon CT (2009). Extracellular histones are major mediators of death in sepsis. Nat Med.

[33] Chaput C, Zychlinsky A (2009). Sepsis: the dark side of histones. Nat Med.

[34] Huang H, Evankovich J, Yan W, Nace G, Zhang L, Ross M, Liao X, Billiar T, Xu J, Esmon CT, Tsung A (2011). Endogenous histones function as alarmins in sterile inflammatory liver injury through Toll-like receptor 9 in mice. Hepatology.

[35] Abrams ST, Zhang N, Manson J, Liu T, Dart C, Baluwa F, Wang SS, Brohl K, Kipar A, Yu W, Wang G, Toh CH (2013). Circulating histones are mediators of trauma-associated lung injury. Am J Respir Crit Care Med.

[36] Fuchs TA, Brill A, Duerschmied D, Schatzberg D, Monestier M, Myers DD, Wrobleski SK, Wakefield TW, Hartwig JH, Wagner DD (2010). Extracellular DNA traps promote thrombosis. Proc Natl Acad Sci U S A.

[37] Fuchs TA, Bhandari AA, Wagner DD (2011). Histones induce rapid and profound thrombocytopenia in mice. Blood.

[38] Xu J, Lupu F, Esmon CT (2010). Inflammation, innate immunity and blood coagulation. Hamostaseologie.

[39] Nakahara M, Ito T, Kawahara K, Yamamoto M, Nagasato T, Shrestha B, Yamada S, Miyauchi T, Higuchi K, Takenaka T, Yasuda T, Matsunaga A, Kakihana Y, Hashiguchi T, Kanmura Y, Maruyama I (2013). Recombinant thrombomodulin protects mice against histone-induced lethal thromboembolism. PLoS One.

[40] Taylor FB, Toh CH, Hoots WK, Wada H, Levi M (2001). Towards definition, clinical and laboratory criteria, and a scoring system for disseminated intravascular coagulation. Thromb Haemost.

[41] Kobayashi N, Maegawa T, Takada M, Tanaka H, Gonmori H (1983). Criteria for diagnosis of DIC based on the analysis of clinical and laboratory findings in 345 DIC patients collected by the Research Committee on DIC in Japan. Bibl Haematol.

[42] Gando S, Iba T, Eguchi Y, Ohtomo Y, Okamoto K, Koseki K, Mayumi T, Murata A, Ikeda T, Ishikura H, Ueyama M, Ogura H, Kushimoto S, Saitoh D, Endo S, Shimazaki S, Japanese Association for Acute Medicine Disseminated Intravascular Coagulation (JAAM DIC) Study Group (2006). A multicenter, prospective validation of disseminated intravascular coagulation diagnostic criteria for critically ill patients: comparing current criteria. Crit Care Med.

[43] Ogura H, Gando S, Iba T, Eguchi Y, Ohtomo Y, Okamoto K, Koseki K, Mayumi T, Murata A, Ikeda T, Ishikura H, Ueyama M, Kushimoto S, Saitoh D, Endo S, Shimazaki S, Japanese Association for Acute Medicine Disseminated Intravascular Coagulation (JAAM DIC) Study Group (2007). SIRS-associated coagulopathy and organ dysfunction in critically ill patients with thrombocytopenia. Shock.

[44] Takemitsu T, Wada H, Hatada T, Ohmori Y, Ishikura K, Takeda T, Sugiyama T, Yamada N, Maruyama K, Katayama N, Isaji S, Shimpo H, Kusunoki M, Nobori T (2011). Prospective evaluation of three different diagnostic criteria for disseminated intravascular coagulation. Thromb Haemost.

[45] Wada H, Wakita Y, Nakase T, Shimura M, Hiyoyama K, Nagaya S, Mori Y, Shiku H (1995). Outcome of disseminated intravascular coagulation in relation to the score when treatment was begun. Thromb Haemost.

[46] Okamoto K, Wada H, Hatada T, Uchiyama T, Kawasugi K, Mayumi T, Gando S, Kushimoto S, Seki Y, Madoiwa S, Asakura H, Koga S, Iba T, Maruyama I, Japanese Society of Thrombosis Hemostasis/DIC subcommittee (2010). Frequency and hemostatic abnormalities in pre-DIC patients. Thromb Res.

[47] Rosenberg RD, Damus PS (1973). The purification and mechanism of action of human antithrombin-heparin cofactor. J Biol Chem.

[48] Warren BL, Eid A, Singer P, Pillay SS, Carl P, Novak I, Chalupa P, Atherstone A, Pénzes I, Kübler A, Knaub S, Keinecke HO, Heinrichs H, Schindel F, Juers M, Bone RC, Opal SM, KyberSept Trial Study Group (2001). High-dose antithrombin in severe sepsis. A randomized controlled trial. JAMA.

[49] Vinazzer H (1989). Therapeutic use of antithrombin III in shock and disseminated intravascular coagulation. Semin Thromb Hemost.

[50] Dzinic L, Marenovic T, Lakic-Trajkovic Z (1991). Clinical study of the therapeutic value of Kybernin in the treatment of antithrombin III deficiency. Med Pregl.

[51] Albert J, Blomqvist H, Gardlund B, Jakobsson J, Svensson J, Blombäck M (1992). Effect of antithrombin concentrate on haemostatic variables in critically ill patients. Acta Anaesthesiol Scand.

[52] Fourrier F, Chopin C, Huart JJ, Runge I, Caron C, Goudemand J (1993). Double-blind, placebocontrolled trial of antithrombin III concentrates in septic shock with disseminated intravascular coagulation. Chest.

[53] Waydhas C, Nast-Kolb D, Gippner-Steppert C, Trupka A, Pfundstein C, Schweiberer L, Jochum M (1998). High-dose antithrombin III treatment of severely injured patients: results of a prospective study. J Trauma.

[54] Baudo F, Caimi TM, de Cataldo F, Ravizza A, Arlati S, Casella G, Carugo D, Palareti G, Legnani C, Ridolfi L, Rossi R, D'Angelo A, Crippa L, Giudici D, Gallioli G, Wolfler A, Calori G (1998). Antithrombin III (ATIII) replacement therapy in patients with sepsis and/or postsurgical complications: a controlled double-blind, randomized, multicenter study. Intensive Care Med.

[55] Eisele B, Lamy M, Thijs LG, Keinecke HO, Schuster HP, Matthias FR, Fourrier F, Heinrichs H, Delvos U (1998). Antithrombin III in patients with severe sepsis. A randomized, placebo-controlled, double-blind multicenter trial plus a meta-analysis on all randomized, placebo-controlled, double blind trials with antithrombin III in severe sepsis. Intensive CareMed.

[56] Hoffmann JN, Muhlbayer D, Jochum M, Inthorn D (2004). Effect of long-term and high-dose antithrombin supplementation on coagulation and fibrinolysis in patients with severe sepsis. Crit Care Med.

[57] Kienast J, Juers M, Wiedermann CJ, Hoffmann JN, Ostermann H, Strauss R, Keinecke HO, Warren BL, Opal SM, KyberSept investigators (2006). Treatment effects of high-dose antithrombin without concomitant heparin in patients with severe sepsis with or without disseminated intravascular coagulation. J Thromb Haemost.

[58] Wiedermann CJ, Kaneider N (2006). A systematic review of antithrombin concentrate use in patients with disseminated intravascular coagulation of severe sepsis. Blood Coagul Fibrinolysis.

[59] Iba T, Saito D, Wada H, Asakura H (2012). Efficacy and bleeding risk of antithrombin supplementation in septic disseminated intravascular coagulation: a prospective multicenter survey. Thromb Res.

[60] Iba T, Saitoh D, Wada H, Asakura H (2014). Efficacy and bleeding risk of antithrombin supplementation in septic disseminated intravascular coagulation: a secondary survey. Crit Care.

[61] Gando S, Saitoh D, Ishikura H, Ueyama M, Otomo Y, Oda S, Kushimoto S, Tanjoh K, Mayumi T, Ikeda T, Iba T, Eguchi Y, Okamoto K, Ogura H, Koseki K, Sakamoto Y, Takayama Y, Shirai K, Takasu O, Inoue Y, Mashiko K, Tsubota T, Endo S (2013). A randomized, controlled, multicenter trial of the effects of antithrombin on disseminated intravascular coagulation in patients with sepsis. Crit Care.

[62] Tagami T, Matsui H, Horiguchi H, Fushimi K, Yasunaga H (2014). Antithrombin and mortality in severe pneumonia patients with sepsis-associated disseminated intravascular coagulation: an observational nationwide study. J Thromb Haemost.

[63] Sakuragawa N, Hasegawa H, Maki M, Nakagawa M, Nakashima M (1993). Clinical evaluation of low-molecular-weight heparin (FR-860) on disseminated intravascular coagulation (DIC)—a multicenter co-operative double-blind trial in comparison with heparin. Thromb Res.

[64] Jaimes F, De La Rosa G, Morales C, Fortich F, Arango C, Aguirre D, Mun˜oz A (2009). Unfractioned heparin for treatment of sepsis: a randomized clinical trial (The HETRASE study). Crit Care Med.

[65] Zarychanski R, Abou-Setta AM, Kanji S, Turgeon AF, Kumar A, Houston DS, Rimmer E, Houston BL, McIntyre L, Fox-Robichaud AE, Hébert P, Cook DJ, Fergusson DA, Canadian Critical Care Trials Group (2015). The efficacy and safety of heparin in patients with sepsis: a systematic review and metaanalysis. Crit Care Med.

[66] Wang C, Chi C, Guo L, Wang X, Guo L, Sun J, Sun B, Liu S, Chang X, Li E (2014). Heparin therapy reduces 28-day mortality in adult severe sepsis patients: a systematic review and meta-analysis. Crit Care.

[67] Iba T, Hashiguchi N, Nagaoka I, Tabe Y, Kadota K, Sato K (2015). Heparins attenuated histone-mediated cytotoxicity in vitro and improved the survival in a rat model of histone-induced organ dysfunction. Intensive Care Med Exp.

[68] Esmon CT (2005). The interactions between inflammation and coagulation. Br J Haematol.

[69] Esmon CT (1987). The regulation of natural anticoagulant pathways. Science.

[70] Grey ST, Tsuchida A, Hau H, Orthner CL, Salem HH, Hancock WW (1994). Selective inhibitory effects of the anticoagulant activated protein C on the responses of human mononuclear phagocytes to LPS, IFN gamma, or phorbol ester. J Immunol.

[71] Abeyama K, Stern DM, Ito Y, Kawahara K, Yoshimoto Y, Tanaka M, Uchimura T, Ida N, Yamazaki Y, Yamada S, Yamamoto Y, Yamamoto H, Iino S, Taniguchi N, Maruyama I (2005). The N-terminal domain of thrombomodulin sequesters high-mobility group-B1 protein, a novel antiinflammatory mechanism. J Clin Invest.

[72] Ito T, Kawahara K, Okamoto K, Yamada S, Yasuda M, Imaizumi H, Nawa Y, Meug X, Shrestha B, Hashiguchi T, Maruyama I (2008). Proteolytic cleavage of high mobility group box 1 protein by thrombin-thrombomodulin complexes. Arterioscler Thromb Biol.

[73] Nagato M, Okamoto K, Abe Y, Higure A, Yamaguchi K (2009). Recombinant human soluble thrombomodulin (ART-123) decreases the plasma HMGB1 levels, while improving the acute liver injury and survival rates in experimental endotoxemia. Crit Care Med.

[74] Saito H, Maruyama I, Shimazaki S, Yamamoto Y, Aikawa N, Ohno R, Hirayama A, Matsuda T, Asakura H, Nakashima M, Aoki N (2007). Efficacy and safety of recombinant human soluble thrombomodulin (ART-123) in disseminated intravascular coagulation: results of a phase III, randomized, double-blind clinical trial. J Thromb Haemost.

[75] Aikawa N, Shimazaki S, Yamamoto Y, Saito H, Maruyama I, Ohno R, Hirayama A, Aoki Y, Aoki N (2011). Thrombomodulin alfa in the treatment of infectious patients complicated by disseminated intravascular coagulation: subanalysis from the phase 3 trial. Shock.

[76] Vincent JL, Ramesh MK, Ernest D, Larosa SP, Pach J, Aikawa N, Hoste E, Levy H, Hirman J, Levi M, Daga M, Kutsogiannis DJ, Crowther M, Bernard GR, Devriendt J, Puigserver JV, Blanzaco DU, Esmon CT, Parrillo JE, Guzzi L, Henderson SJ, Pothirat C, Mehta P, Fareed J, Talwar D, Tsuruta K, Gorelick KJ, Osawa Y, Kaul I. A randomized, double-blind, placebo-controlled, phase 2b study to evaluate the safety and efficacy of recombinant human soluble thrombomodulin, ART-123, in patients with sepsis and suspected disseminated intravascular coagulation. Crit Care Med. 2013;41:2069–79. 10.1097/CCM.0b013e31828e9b03 23979365

[77] Tagami T, Matsui H, Horiguchi H, Fushimmi K, Yasunaga H (2015). Recombinant human soluble thrombomodulin and mortality in severe pneumonia patients with sepsis-associated disseminated intravascular coagulation: an observational nationwide study. J Thromb Haemost.

[78] Murata A, Okamoto K, Mayumi T, Muramatsu K, Matsuda S (2014). The recent time trend of outcomes of disseminated intravascular coagulation in Japan: an observational study based on a national administrative database. J Thrombosis Thrombolysis.

[79] Murata A, Okamoto K, Mayumi T, Muramatsu K, Matsuda S (2015). Observational study to compare antithrombin and thrombomodulin for disseminated intravascular coagulation. Int J Clin Pharm.

[80] Murata A, Okamoto K, Mayumi T, Muramatsu K, Matsuda S. Recent change in treatment of disseminated intravascular coagulation in Japan: an epidemiological study based on a National Administrative Database. Clin Appl Thromb Hemost. 2016;22:21-27. 10.1177/1076029615575072 25736054

